# Seasonal variation of peptic ulcer disease, peptic ulcer bleeding, and acute pancreatitis: A nationwide population-based study using a common data model

**DOI:** 10.1097/MD.0000000000025820

**Published:** 2021-05-28

**Authors:** Jin Young Yoon, Jae Myung Cha, Ha Il Kim, Min Seob Kwak

**Affiliations:** Department of Internal Medicine, Division of Gastroenterology, Kyung Hee University Hospital at Gang Dong, Seoul, Republic of Korea.

**Keywords:** acute pancreatitis, peptic ulcer, peptic ulcer bleeding, seasonality, seasonal variation

## Abstract

Although gastrointestinal diseases are reported at various times throughout the year, some particular seasons are associated with a higher incidence of these diseases. This study aimed to identify the seasonal variations of peptic ulcer (PU), peptic ulcer bleeding (PUB), and acute pancreatitis (AP) in South Korea.

We conducted a retrospective, observational cohort study of all subjects aged >18 years between 2012 and 2016 using the Health Insurance Review and Assessment-National Patient Samples database, previously converted to the standardized Observational Medical Outcomes Partnership-Common Data Model. We assessed the overall seasonal variations of PU, PUB, and AP and further analyzed seasonal variations according to age and sex subgroups.

In total, 14,626 patients with PU, 3575 with PUB, and 9023 with AP were analyzed for 5 years. A clear seasonal variation was noted in PU, with the highest incidence rate during winter, the second highest during spring, the third highest during summer, and the lowest incidence during autumn for 5 years (*P* < .001). PUB also showed significant seasonal fluctuations, with winter peak for 4 years, except 1 year, which had a spring peak (*P* < .001). However, AP showed no clear seasonal variations (*P* = .090). No significant differences in the seasonal variation of PU, PUB, and AP were observed according to sex and age subgroups (<60 years vs ≥60 years).

Seasonal variation of PU and PUB should be considered when determining allocation of available health care resources.

## 1 Introduction

Seasonality is a phenomenon relating to incidence pattern in the epidemiology of several diseases.^[1]^ The knowledge of disease seasonality contributes to not only understand etiology and pathogenesis but also develop effective strategies for preventing these diseases and help policymakers plan for appropriate optimal distribution of healthcare resources and risk stratification of patients during the peak season.^[2]^ The concept of seasonal variations has been well established in the previous literatures for cardiovascular, pulmonary, and rheumatologic diseases,^[3–5]^ and particularly in disease entities requiring prohibitive medical resources such as sepsis, stroke, and cardiac arrest.^[6–8]^

Gastrointestinal (GI) diseases have been reported to occur throughout the year, but show some seasonal variation.^[1]^ Analyses for seasonality of GI diseases have been dedicated to diseases that acute or intensive care is pivotal treatment such as peptic ulcer bleeding (PUB), peptic ulcer (PU), and acute pancreatitis (AP).^[9–13]^ Since PUB typically requires an emergent endoscopic procedure for bleeding control, prediction of its incidence is important to prepare the presence of backup personnel with sufficient capacity during the peak season in order to provide the best treatment. As most patients with AP are hospitalized for intensive care with aggressive hydration and nutritional support, predicting the demand for hospital resource is crucial for optimizing patient care. Unfortunately, previous studies on the seasonal variation of PUB, PU, and AP have been performed in only a few Western and Asian countries and inconclusive.^[9–22]^

To the best of our knowledge, no study has comprehensively examined the seasonal variation of PU, PUB, and AP in the Korean population, despite the fact that the seasonality of South Korea is significant, with a great annual fluctuation in climate. Therefore, this study aimed to identify the seasonal variations of PU, PUB, and AP in South Korea using a nationwide population-based database and a common data model (CDM).

## 2 Materials and methods

### 2.1 Data source

We used the Health Insurance Review and Assessment-National Patient Samples (HIRA-NPS) database from 2012 to 2016, previously converted to the standardized Observational Medical Outcomes Partnership (OMOP)-CDM. HIRA is a repository of claims data collected in the process of reimbursing healthcare providers with fee-for-services covering all people in South Korea under the universal coverage system.^[23]^ It contains comprehensive information pertaining to healthcare services, such as treatments, medications, procedures, and diagnoses, for almost 50 million beneficiaries.^[23]^ HIRA-NPS accounts for 3% of the national patient sample (about 1,000,000) in a year, which was extracted using a stratified randomized sampling method according to sex and age group. It includes about 13% (approximately 700,000) of inpatient data and 1% (approximately 400,000) of outpatient data, including all data related to medical claims and prescriptions for 1 year. For the extraction method, HIRA-NPS adopted stratified sampling, a probabilistic sample extraction method. Under the assumption of acceptable sampling error range and normal distribution, the standard deviation and the sample size were calculated for the optimal size of HIRA-NPS. HIRA-NPS has the advantages of accessibility and ease of handling over the entire HIRA data. However, it is difficult to characterize or analyze the data from HIRA-NPS and each hospital (especially for international hospital data) in the same way or using the same tools because healthcare data sets are stored in databases that are built using a wide variety of data models and, often, local terminologies.^[24,25]^ An analysis across multiple disparate databases must either tailor the analysis to accommodate each of the underlying data models and terminologies or convert the databases to a CDM.^[25]^

Recently, HIRA-NPS was converted to the OMOP-CDM in Korea, which is designed to include all observational health data elements to support the generation of reliable scientific evidence.^[24,25]^ Variable terms for HIRA-NPS, which are typically expressed in nonstandard terms, were standardized into standard concepts using mapping. As HIRA data were initially recorded using International Classification of Diseases, version 9 codes, they were mapped to systemized nomenclature for medicine-clinical terms codes in CDM data.^[25]^ In OMOP-CDM, disparate coding systems can be harmonized – with minimal information loss – to a standardized vocabulary. As the OMOP-CDM version of HIRA-NPS database is provided as an open source in South Korea, it has advantages over original HIRA-NPS database, which was limited by a preapproval for use, a usage fee, and a limited use through a predetermined platform for on-line or off-line use.^[24,25]^

### 2.2 Study design and cohort definition

We conducted a retrospective, observational cohort study of all subjects aged >18 years between January 1, 2012 and December 31, 2016. We described the clinical characteristics of the study population with each condition and their seasonal variations by index year. Seasons were divided as follows: spring (March–May), summer (June–August), autumn (September–November), and winter (December–February). In order to examine changes across the seasons, we identified the proportion of patients with PU, PUB, or AP in each season among the total number of patients in the index year. We assessed variables including age, gender, and Charlson index, and analyzed the seasonal pattern in the stratified demographics, such as age groups (young age group, 18–59 years vs old age group, ≥60 years) and sex (men vs women). The Charlson Index was used to predict the 10-year mortality for a patient who may have a range of comorbid conditions such as heart disease, diabetes, AIDS, or cancer.^[26,27]^ A currently commonly used version of this index identified a total of 22 comorbidities that are associated with mortality and the final score is obtained via the summation of applicable points and ranges from 0 (no disease burden) to 29 (maximal disease burden).^[27]^

The PU cohort included patients initially diagnosed with gastric or duodenal ulcer after an inpatient or emergency department (ED) visit. The PUB cohort included patients initially diagnosed during inpatient or ED visit with upper GI bleeding caused by gastric or duodenal ulcer. Patients with variceal bleeding were excluded from the PUB cohort. The AP cohort included patients initially diagnosed with AP. Only patients aged >18 years were included in all 3 cohorts. All captured events were defined as a condition occurrence for the first time in the person's history. In addition, we limited initial events to earliest event per person during study period. Concept identification and code for concept sets (PU, PUB, AP, inpatient, or ED visit) are presented in Table S1, Supplemental Digital Content. This study was approved by the Institutional Review Board of our study institution (KHNMC IRB 2020-04-032).

### 2.3 Statistical analysis

The OMOP-CDM analysis tools are embedded in the interactive analysis platform ATLAS. ATLAS version 2.7.2 was used, and we analyzed the platform of FEEDER-NET, a health big-data platform based on the OMOP-CDM supported by the Korean National Project. Continuous variables, presented as means ± standard deviations for clinical data, were compared using 2-sample *t*-tests. Categorical variables are presented as numbers and percentages and were compared using chi-square tests. For the analysis of seasonal variation, we identified the proportion of patients with each disease in each season among the total number of patients in the index year. To detect any deviation form a uniform seasonal distribution of the frequency of diseases by site throughout the year, repeated-measures ANOVA of serial variances were used. All *P*-values were 2-tailed, and values less than .05 were considered statistically significant.

## 3 Results

### 3.1 Clinical characteristics of study population

Table 1 shows the clinical characteristics of the study population with PU by index year. In total, 14,626 patients with PU were analyzed: 2946 patients in 2012; 2842 patients in 2013; 2765 patients in 2014; 2865 patients in 2015; and 3208 patients in 2016. No temporal trend was detected for the average age in each year. In age subgroups, the overall distribution of patients with PU was 45.1% in the old age (≥60 years) group and 54.9% in the young age (18–59 years) group. In sex subgroups, women were predominant throughout the entire study period (55.9% vs 44.1%). There were no significant changes in the proportion of age and sex of the subjects in each year (*P* = .761 and *P* = .977, respectively). The Charlson index revealed a gradual decreasing tendency (*P* = .004) from 3.3 ± 2.9 in 2012 to 3.1 ± 2.6 in 2016.

**Table 1:** Clinical characteristics of study population with peptic ulcer disease by index year.

	2012	2013	2014	2015	2016
Number of patients	2946	2842	2765	2865	3208
Age (yr), m ± SD	57.0 ± 16.8	57.0 ± 16.9	55.4 ± 16.6	58.1 ± 16.8	57.7 ± 17.5
Age (yr) group, n (%)					
18–59 yr	1603 (54.4)	1599 (56.3)	1646 (59.5)	1483 (51.8)	1698 (52.9)
≥60 yr	1343 (45.6)	1243 (43.7)	1119 (38.9)	1382 (48.2)	1510 (47.1)
Sex, n (%)					
Men	1277 (43.4)	1299 (45.7)	1226 (44.3)	1303 (45.5)	1352 (42.1)
Women	1669 (56.6)	1543 (54.3)	1539 (55.7)	1562 (54.5)	1856 (57.9)
Charlson index^∗^, m ± SD	3.3 ± 2.9	3.3 ± 2.8	3.3 ± 2.8	3.2 ± 2.7	3.1 ± 2.6

A total of 3575 patients with PUB were included during the study period: 759 patients in 2012; 777 patients in 2013; 685 patients in 2014; 682 patients in 2015; and 672 patients in 2016 (Table 2). Overall, patients with PUB were likely to be older (57.4% vs 42.6%) and male (60.2% vs 39.8%). The proportion of sex in each year did not significantly change throughout the study period (*P* = .953). Regarding the incidence of PUB, the proportion of male subjects gradually declined from 62.9% in 2014 to 56.3% in 2016; however, this change was not significant (*P* = .841). No temporal trend was detected regarding the Charlson index (*P* = .317).

**Table 2:** Clinical characteristics of study population with peptic ulcer bleeding by index year.

	2012	2013	2014	2015	2016
Number of patients	759	777	685	682	672
Age (yr), m ± SD	61.3 ± 16.1	62.3 ± 16.4	61.3 ± 16.2	63.1 ± 16.3	63.0 ± 17.4
Age (yr) group, n (%)					
18–59 yr	342 (45.1)	316 (40.7)	306 (44.7)	279 (40.9)	281 (41.8)
≥60 yr	417 (54.9)	461 (59.3)	379 (55.3)	403 (59.1)	391 (58.2)
Sex, n (%)					
Men	463 (61.0)	485 (62.4)	431 (62.9)	396 (58.1)	378 (56.3)
Women	296 (39.0)	292 (37.6)	254 (37.1)	286 (41.9)	294 (43.7)
Charlson index^∗^, m ± SD	4.1 ± 3.3	4.3 ± 3.2	4.3 ± 3.4	4.3 ± 3.1	4.1 ± 3.1

In total, 9023 patients with AP were analyzed during the study period: 1227 patients in 2012; 1278 patients in 2013; 1565 patients in 2014; 1883 patients in 2015; and 3070 patients in 2016 (Table 3). In a subanalysis according to age groups, AP diagnosis is more likely in younger than in older individuals (53.3% vs 46.7%). Overall, sex distribution of patients with AP was 53.3% in men and 46.7% in women. The proportion of age and sex in each year did not significantly change throughout the study period (*P* = .850 and *P* = .844, respectively). The average Charlson index was similar for the first 4 years; meanwhile, there was a significant decrease in 2016 (*P* < .001).

**Table 3:** Clinical characteristics of study population with acute pancreatitis by index year.

	2012	2013	2014	2015	2016
Number of patients	1227	1278	1565	1883	3070
Age (yr), m ± SD	57.2 ± 16.9	57.2 ± 6.7	56.2 ± 16.5	58.0 ± 17.0	55.4 ± 18.2
Age (yr) group, n (%)					
18–59 yr	682 (55.6)	701 (54.9)	909 (58.0)	982 (52.2)	1785 (58.1)
≥60 yr	545 (44.4)	577 (45.1)	656 (42.0)	901 (49.8)	1,285 (41.9)
Sex, n (%)					
Men	690 (56.2)	712 (55.7)	859 (54.9)	1035 (55.0)	1516 (49.4)
Women	537 (43.8)	566 (44.3)	706 (45.1)	848 (45.0)	1554 (50.6)
Charlson index^∗^, m ± SD	3.3 ± 2.9	3.3 ± 2.9	3.3 ± 3.0	3.3 ± 2.9	2.9 ± 2.9

### 3.2 Seasonal variations

**Figure 1:**
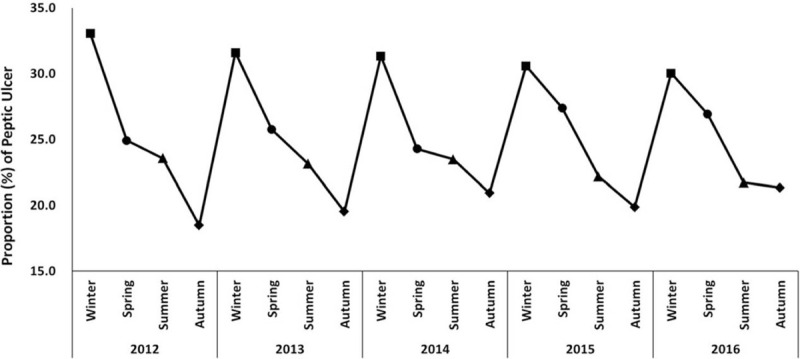
Seasonal variation in patients presenting with peptic ulcer disease by index year.

During the 5-year study period, seasonal variations were found in incidence of PU, with the highest incidence of PU during winter, followed by spring and summer, and a nadir during autumn (Fig. 1, *P* < .001). Peak values during winter gradually reduced from 33.1% in 2012 to 30.0% in 2016. In addition, values of the second peak during spring varied between 24.3% and 27.4%, those of the third peak during summer varied between 21.7% and 24.6%, and those of the last peak during autumn varied between 18.5% and 21.3%. A steep rise in PU incidence was noted from autumn to winter. Figure S1, Supplemental Digital Content showed no significant differences in seasonal variation of PU in age and sex subgroups (*P* = .111 and *P* = .074, respectively).

**Figure 2:**
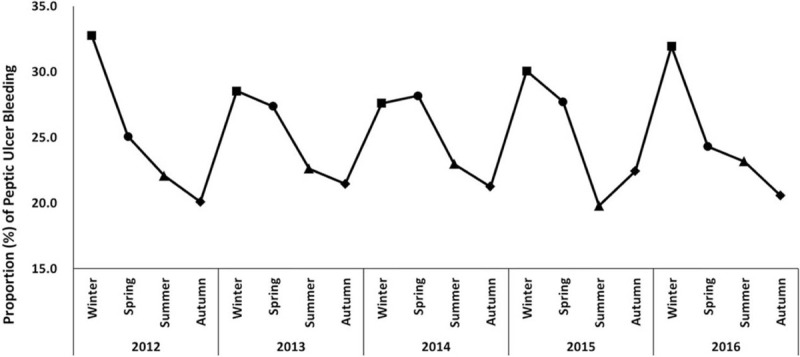
Seasonal variation in patients presenting with peptic ulcer bleeding by index year.

Seasonal variations were also found in incidence of PUB, with the highest incidence during winter and the second peak during spring for 4 years (2012, 2013, 2015, and 2016) (Fig. 2, *P* < .001). However, in 2014, the highest peak was observed during spring and the second peak during winter. The distribution of the winter peak was the highest in 2012 (32.8%) and lowest 2013 (28.5%). Nadir seasons were noted mainly during autumn, with an incidence of 19.8% to 21.5%. Figure S2, Supplemental Digital Content showed no significant differences in the seasonal variation of PUB in age and sex subgroups (*P* > .999 and *P* = .305, respectively).

**Figure 3:**
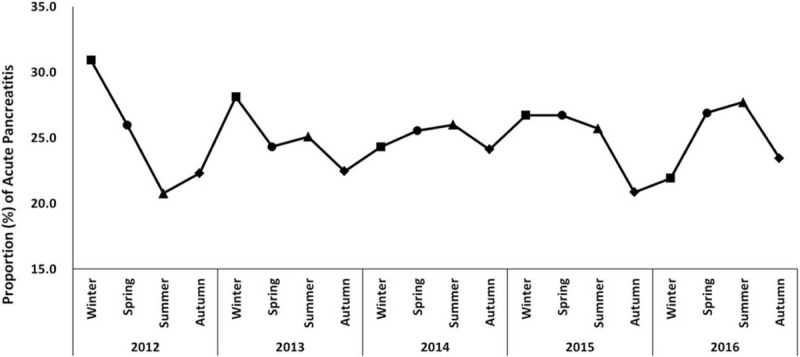
Seasonal variation in patients presenting with acute pancreatitis by index year.

No seasonal variations were noted for incidence of AP, as there was no remarkable peak and various seasonal peaks in the AP occurrence during study period (Fig. 3, *P* = .090). Figure S3, Supplemental Digital Content showed no significant differences in seasonal variation of AP according to age and sex subgroups (*P* = .608 and *P* = .217, respectively).

## 4 Discussion

Understanding seasonal variation of GI diseases may help physicians plan for appropriate healthcare distribution and seasonal availability of resources for the peak season. Previous studies on this issue were reported from other countries but showed various results (Table 4). In this study, we evaluated the seasonal variation of PU, PUB, and AP in Korea using a well-defined HIRA-NPS database over a 5-year period. To our knowledge, this is the first study to evaluate seasonal variation with a recent nationwide database in an Asian population. The most important finding of the present study is that a clear seasonal variation was observed in PU, with the highest incidence rate during winter and the lowest during autumn, during a 5-year period. PUB was also associated with significant seasonal fluctuations, including winter peak for 4 years; however, AP showed no seasonal variations. In our study, there was a gradual reduction in winter peak values of PU, from 33.1% in 2012 to 30.0% in 2016. Gradual reduction of PU incidence may be explained by increased use of proton pump inhibitors (PPIs) in the recent years.^[28,29]^

**Table 4:** Seasonal variation in the onset of peptic ulcer (PU), peptic ulcer bleeding (PUB), and acute pancreatitis (AP) in previously published studies 2000s.

Disease	Author/setting	Sample size	Data base	Time period	Seasonal peak
Peptic ulcer					
	Cherian JV et al, 2010 (India)^[14]^	Unknown	Single center	1989–2004	Winter-Spring
	Xirasagar S et al, 2007 (Taiwan)^[9]^	160,510	Nationwide database	1997–2003	Winter
	Manfredini R et al, 2010 (Italy)^[15]^	976	Single center	1998–2005	Autumn, Winter, and Spring
	Kanotra R et al, 2016 (USA)^[10]^	351,921	Nationwide database	2000–2011	Spring
	Current study, 2021 (Korea)	14,626	Nationwide database	2012–2016	Winter
Peptic ulcer bleeding					
	Lopez-Cepero et al, 2005 (Spain)^[11]^	499	Single center	1998–2001	No seasonal variation
	Sezgin O et al, 2007 (Turkey)^[16]^	237	Single center	2001–2005	Spring
	Du T et al, 2010 (China)^[17]^	2580	Multicenter in area	2005–2007	From December to April
	Lenzen H et al, 2017 (Germany)^[18]^	304	Single center	2007–2012	Winter
	Yuan XG et al, 2015 (China)^[19]^	176	Single center	2009–2010	Extreme cold climate
	Current study, 2021 (Korea)	3575	Nationwide database	2012–2016	Winter
Acute pancreatitis					
	Gallerani M et al, 2004 (Italy)^[12]^	549	Single center	1998–2002	Spring
	Roberts SE et al, 2013 (Wales, UK)^[20]^	10,589	Multicenter in area	1999–2010	Winter (Christmas and New Year weeks)
	Bertilsson S et al, 2017 (Sweden)^[21]^	1457	Single center	2003–2012	No seasonal variation
	Guarino M et al, 2020 (Italy)^[13]^	1883	Single center	2003–2017	Summer
	Wu D et al, 2017 (China)^[22]^	1780	Single center	2009–2014	Spring and Autumn
	Current study, 2021 (Korea)	9023	Nationwide database	2012–2016	No seasonal variation

Until recently, most studies have reported seasonal variation of PU, with the peak incidence during winter.^[1]^ However, among these studies, there were some differences in the observed seasonality pattern. For example, Indian or Taiwan studies have shown increased PU incidence during winter and spring,^[14,30,31]^ whereas Israeli, Norwegian, or Italian studies have shown autumn and winter peaks.^[32–34]^ In our study, we observed a peak incidence of PU during winter, followed by spring, summer, and autumn, with a steep rise between autumn and winter. In terms of demographic characteristics, early studies have reported that PU and PUB is more prevalent in elderly patients, which maybe because of the existence of comorbidities and increased intake of nonsteroidal anti-inflammatory drugs (NSAIDs).^[35,36]^ Our study also showed that PUB was significantly more prevalent in elderly male patients, which is consistent with the results of previous studies.^[37–39]^ However, in our study, PU occurred more frequently in young female patients, which differs from the results of previous studies.^[10,19]^ This unique finding could be explained by increased use of NSAIDs in women than men in Korea during the study period (Table S2, Supplemental Digital Content).

Although the underlying mechanism of these seasonal variations remains unclear, the seasonality of PU and PUB may be explained by 3 factors: (1) physiological changes from climate; (2) ulcerogenic medications (such as NSAIDs, aspirin, and corticosteroids), or (3) *Helicobacter pylori* (*H pylori*) infection. Yuan et al have reported that the thickness of gastric mucus becomes significantly thinner in an extremely cold climate than in an extremely hot climate, which could weaken the defense mechanisms of the gastric mucosa.^[19]^ In addition, expression of heat shock protein 70 is reduced in cold climate; the protein has a key role in rescue from apoptosis, facilitation of mucosal healing, and protection against harmful substances.^[19,40]^ Physical stress caused by cold climate and rapid climate change could cause secretion of adrenaline and noradrenalin by sympathetic nerve stimulation.^[41]^ This response could accelerate secretion of endothelin, which causes contraction of blood vessels in the gastroduodenal mucosa and deceleration of mucosal blood flow, which may result in development of PU.^[9]^ The seasonality of PU and PUB may also be influenced by fluctuations in the use of ulcerogenic drugs. Intake of NSAIDs, which is known as the most common risk factor of PU and PUB, increases during cold climate due to aggravation of arthritis symptoms in patients with rheumatoid arthritis or osteoarthritis.^[5,9,14]^ Respiratory diseases, such as chronic obstructive pulmonary disease or asthma, are often aggravated during winter and result in increased corticosteroid use.^[4]^ In addition, acute and prolonged cold exposure may increase the incidence of cardiovascular disease requiring aspirin use.^[3]^ To date, there are inconsistent results in the literature regarding the correlation between the seasonality of PU and frequency of *H pylori* infection. No significant difference has been reported in the prevalence of *H pylori* infection among hot and cold climates.^[33,42,43]^ However, in 2 studies on patients with PU, the frequency of *H pylori* infection significantly increased during winter and decreased during summer.^[44,45]^

In our study, no seasonal variation was observed in incidence of AP during the study period. Fluctuations in alcohol consumption, a major risk factor of AP,^[21]^ could have potential effects on the occurrence of AP. Therefore, the incidence of AP may have been influenced by the holiday season rather than the climate change.^[20]^ Furthermore, occurrence of AP has a complex correlation with many factors in addition to alcohol consumption, including quantity of alcohol, drinking interval, comorbidity, and drug history.^[21,46]^ Currently, reports of seasonal variation of AP are lacking; seasonal variation of AP has been reported in studies from Finland, Wales, and China,^[20,22,47]^ but in studies from Germany and Sweden.^[21,48]^ Italian studies have also reported a seasonal variation of AP; however, the peak season was different in different studies from the same region in Italy.^[12,13]^ Nevertheless, no seasonal variation of AP was observed in our study.

The strength of our study is that it provides insight into the current seasonal variation for PU, PUB, and AP from 2012 to 2016. Most previous studies were conducted in Western countries before the increased use of PPI therapy; therefore, our study is unique – it presents results for seasonal variation in the PPI era.^[18]^ We used a nationwide, large scale database with long-term assessment throughout a 5-year period. This increased the statistical power of the results, despite the need to divide the subjects for seasonality analysis. Additionally, South Korea has 4 distinct seasons, which is particularly pertinent for identifying disease seasonality. Nevertheless, this study also has some limitations. Data collection was retrospective and data quality issues were inevitable in the conversion of HIRA data to the CDM database. However, excellent data quality has been reported in recent studies using the CDM database.^[49,50]^ As the HIRA-NPS includes claim-based data, it is difficult to assess detailed classification of disease characteristics, information on comorbidities, causes of mortality, geographical distribution, and medication history of enrolled patients.

In conclusion, a strong seasonal variation was noted in PU, with the highest incidence rate during winter and the lowest during autumn. PUB also showed seasonal fluctuations; however, AP showed no seasonal variations in our study. Seasonal variation of PU and PUB should be considered when determining allocation of available health care resources.

## Author contributions

**Conceptualization:** Jae Myung Cha.

**Data curation:** Jin Young Yoon, Jae Myung Cha.

**Formal analysis:** Jin Young Yoon.

**Methodology:** Jae Myung Cha, Ha Il Kim, Min Seob Kwak.

**Project administration:** Ha Il Kim, Min Seob Kwak.

**Supervision:** Jae Myung Cha.

**Validation:** Ha Il Kim.

**Writing – original draft:** Jin Young Yoon.

**Writing – review & editing:** Jin Young Yoon, Jae Myung Cha.

## Supplementary Material

Supplemental Digital Content

## Supplementary Material

Supplemental Digital Content

## Supplementary Material

Supplemental Digital Content

## Supplementary Material

Supplemental Digital Content

## Supplementary Material

Supplemental Digital Content
