# Supplementary material for: Seasonal variation of peptic ulcer disease, peptic ulcer bleeding, and acute pancreatitis: A nationwide population-based study using a common data model
Source: Medicine (Baltimore). 2021 May 28;100(21):e25820. doi: 10.1097/MD.0000000000025820 (PMC8154390; doi:10.1097/MD.0000000000025820)
Supplement: Supplemental Digital Content [file medi-100-e25820-s001.docx]

**Supplementary Table 1.** Concept identification and code for concept sets

| Concept sets | Concept identification | Concept code |
| --- | --- | --- |
| Acute pancreatitis | 199074 | 197456007 |
| UGI bleeding | 4291649 | 37372002 |
| Peptic ulcer bleeding | 4206466, 4271696, 4266523, 4049466  46269893, 4099014, 4247008,  4174044, 4294973, 4211001, 4289830,  4232181, 4006994, 4046500, 4169592,  4231580, 4336230, 4027729 | 55617001, 64121000, 62366003, 15902003  108604100119105, 27281001, 61300005  49232000, 76181002, 57246001, 36975000,  89469000, 111353003, 12274003, 48974009,  89748001, 86895006, 12847006 |
| Peptic ulcer disease | 4027663 | 13200003 |
| Inpatient or ED visit | 9201, 4163685 | IP, 4525004 |

UGI, upper gastrointestinal; ED, emergency department; IP, inpatient
