# Supplementary material for: Seasonal variation of peptic ulcer disease, peptic ulcer bleeding, and acute pancreatitis: A nationwide population-based study using a common data model
Source: Medicine (Baltimore). 2021 May 28;100(21):e25820. doi: 10.1097/MD.0000000000025820 (PMC8154390; doi:10.1097/MD.0000000000025820)
Supplement: Supplemental Digital Content [file medi-100-e25820-s005.docx]

**Supplementary Table 2**. The number of prescriptions for non-steroidal anti-inflammatory drug (NSAID) use by index year in whole population from HIRA-NPS

|  | 2012 | 2013 | 2014 | 2015 | 2016 |
| --- | --- | --- | --- | --- | --- |
| Number of patients | 153,326 | 163,238 | 167,959 | 177,568 | 190,463 |
| Age (years) group, n (%) |  |  |  |  |  |
| 18–59 years | 76,292 | 80,397 | 90,120 | 86,166 | 91,839 |
| ≥60 years | 77,034 | 82,841 | 77,839 | 91,402 | 98,624 |
| Sex, n (%) |  |  |  |  |  |
| Men | 70,688 | 75,463 | 77,876 | 82,552 | 88,387 |
| Women | 82,638 | 87,775 | 90,083 | 95,016 | 102,076 |

HIRA-NPS, Health Insurance Review and Assessment-National Patient Samples
